# Serum Pepsinogen Values in Japanese Junior High School Students With Reference to *Helicobacter Pylori* Infection

**DOI:** 10.2188/jea.JE20180119

**Published:** 2020-01-05

**Authors:** Masumi Okuda, Yingsong Lin, Katsuhiro Mabe, Mototsugu Kato, Takako Osaki, Ryosuke Miyamoto, Akihisa Okumura, Shigeru Kamiya, Shogo Kikuchi

**Affiliations:** 1 Department of Pediatrics, Aichi Medical University School of Medicine, Aichi, Japan; 2 Department of General Medicine and Community Health Science, Hyogo College of Medicine, Hyogo, Japan; 3 Department of Public Health, Aichi Medical University, School of Medicine, Aichi, Japan; 4 Department of Cancer Preventive Medicine, Graduate School of Medicine, Hokkaido University, Hokkaido, Japan; 5 Division of Endoscopy, Hokkaido University Hospital, Hokkaido, Japan; 6 Department of Infectious Diseases, Kyorin University School of Medicine, Tokyo, Japan

**Keywords:** students, urine antibody, serum antibody, serum pepsinogen

## Abstract

**Background:**

Distributions of serum pepsinogen (PG) values were assessed in *Helicobacter pylori*-infected and non-infected junior high school students (aged 12–15 years) in Japan.

**Methods:**

All junior high school students (1,225 in total) in Sasayama city, who were basically healthy, were asked to provide urine and serum samples, which were used to measure urine and serum *H. pylori* antibodies using ELISA kits and PG values. The subjects, whose urine and serum antibodies were both positive, were considered *H. pylori* infected.

**Results:**

Of the 187 subjects who provided urine and blood samples, 8 were infected, 4 had discrepant results, 4 had negative serum antibody titers no less than 3.0 U/ml, and 171 were non-infected. In the *H. pylori* non-infected subjects, the median PG I and PG II values and PG I to PG II ratio (PG I/II) were 40.8 ng/mL, 9.5 ng/mL, and 4.4, respectively, whereas in the infected subjects, these values were 55.4 ng/mL, 17.0 ng/mL, and 3.3, respectively (each *P* < 0.01). In the non-infected subjects, PG I and PG II were significantly higher in males than in females (*P* < 0.01).

**Conclusions:**

The PG I and PG II values were higher, and the PG I/II was lower in *H. pylori* infected students than in non-infected students. In *H. pylori* non-infected students, males showed higher PG I and PG II values than females. The distributions of PG values in junior high school students differed from those in adults.

## INTRODUCTION

Pepsinogen is a precursor of pepsin, and human gastric mucosa cells produce two immunochemically distinct forms of PG.^1^ PG I is secreted by the chief and mucus neck cells in the gastric fundic glands, and PG II is produced by these cells and by the cardiac, pyloric, and Brunner’s glands in the gastric cardia and antrum and proximal duodenum.^2^ PG reflects gastric mucosal atrophy and inflammation, both of which *Helicobacter pylori* (H. pylori) infection provokes. Inflammation upregulates production of both PG I and PG II in gastric mucosal cells and increases the amount discharged to serum, where elevation of PG II is usually larger so that the PG I/II ratio declines. With the progression of atrophy, numbers of gastric mucosal cells producing PG I and PG II decease. As the decrease of cells producing PG I is more crucial, the PG I/II ratio declines with the progression of atrophy.^3^^–^^6^ In adults, PG values were used as a marker of gastric mucosal atrophy that is strongly related to gastric cancer risk.^7^^–^^9^ Recently, criteria of PG values to distinguish subjects with and without *H. pylori* infection have been proposed because PG values differ depending on the infection among adult subjects.^10^ Adults with *H. pylori* infection showed elevated PG I and PG II values and reduced PG I to PG II ratios.^11^

*H. pylori* infection causes lesions in most infected high school students (aged 15–18 years), including nodular/atrophic gastritis and duodenal erosion/ulcer,^12^ and a subset of infected subjects develop gastric cancer in the future.^13^^,^^14^

In a previous study with 454 asymptomatic junior high school students aged 12–15 years in Japan^15^ and another study analyzing sera from 300 asymptomatic Japanese children less than 15 years old,^16^ serum *H. pylori* antibody-positive children showed elevated PG I and PG II, and reduced PG I/II compared with the seronegative children. Thus, PG values can be used to diagnose *H. pylori* infection status in junior high school students, who are usually aged 12–15 years.

Nonetheless, it is still unclear whether distributions of PG values in junior high school students are similar to ones in adults with reference to *H. pylori* infection status. The previous studies did not focused on these points. The aim of this study was to assess the distributions of PG values in *H. pylori* infected and non-infected junior high school students in Japan.

## METHODS

This study was approved by the institutional review boards of Hyogo College of Medicine.

### Subjects and collection of samples

The sample collection was conducted in Sasayama city, which is approximately 60 km north-north-west of Osaka. The population of Sasayama city is approximately 42,000, and the economy relies on agriculture and tourism. In 2012, all 1,225 students attending any of the 6 junior high schools in Sasayama city were invited to participate in the present study. They were healthy students aged 12–15 years and were asked to provide urine and serum samples. The invitation was distributed through the schools. Collection of the samples was performed in several community centers after school or on holidays. The participants went there with their parent or guardian, who were informed of the study and gave the written consent. Urine and blood samples were assayed using *H. pylori* IgG antibody kits. In addition, PG I and PG II levels were measured in the serum samples. The results of the tests were sent to the parents or guardians via the postal system.

### Evaluation of *H. pylori* IgG antibodies (antibody tests) and PG I and II

For the urine antibody tests, single-void urine samples were obtained. Urinary IgG antibodies to *H. pylori* were determined using a urine-HpELISA kit (URINELISA, Otsuka Pharmaceuticals Co., Ltd., Tokyo, Japan). Cut-off index (CI) values (urine antibody titer) ≥1.0 were considered positive for *H. pylori*, and those <1.0 were considered negative. Previous studies showed that the cutoff value gave 91.9% sensitivity and 91.6% specificity,^17^ or a 97.6% sensitivity and a 96.5% specificity^18^ for junior high school students.

For the serum antibody tests, serum *H. pylori* IgG antibody was quantified using a serum-HpELISA kit (E-plate EIKEN *H. pylori*, Eiken Chemical Co., Ltd., Tokyo, Japan). According to the manufacturer’s instruction, serum antibody titers ≥10.0 U/mL and titers <10.0 U/mL were classified as positive and negative for *H. pylori*, respectively. The cutoff value gave 91.2% sensitivity and 97.4% specificity for children aged 0–17 years.^19^ Recently, it has been demonstrated that many subjects with current or past infection have serum antibody titers ≥3.0 U/mL and <10.0 U/mL.^20^ Subjects with such serum antibody titers (negative with relatively high titers) were separated from other subjects whose serum antibody titers were <3.0 U/mL (negative with relatively low titers) and were not included in the *H. pylori* non-infected subjects.

Quantification of PG I and II levels was conducted using the CLIA method (Architect Pepsinogen I, II; Abbott Japan Corp., Tokyo, Japan). Levels of PG I and PG II and the ratio of PG I to PG II were evaluated between positive and negative serum antibody tests. In the non-infected subjects, effect of age (among three school years aged 12–13, 13–14, and 14–15 years) and gender was evaluated.

### Statistical analyses

Statistical analyses were preformed using R version 3.4.1 (R Foundation for Statistical Computing, Vienna, Austria). Differences of PG values were tested using the non-parametric method: the Mann-Whitney U test for comparisons of two groups and the Kruskal-Wallis Chi-squared for comparisons of three groups.

## RESULTS

**Figure 1.**
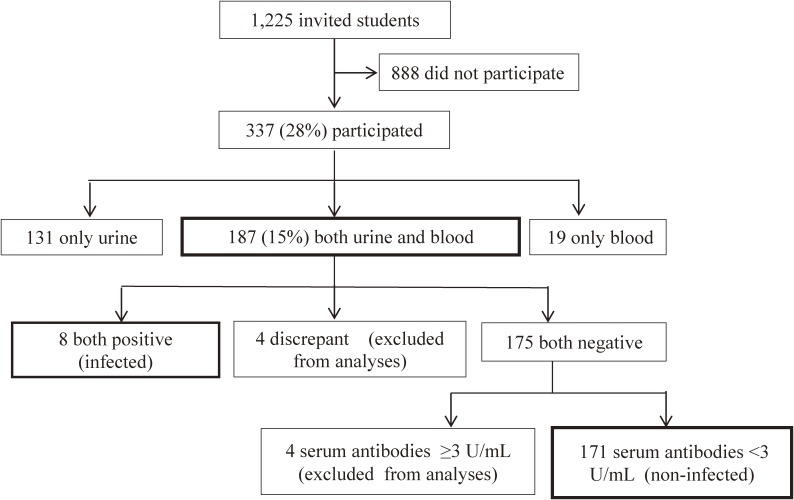
A flow chart for the selection of study subjects

In this study, 337 (28% of those invited) participated, of whom 131, 187, and 19 provided only urine, urine and blood, and only blood samples, respectively (Figure 1). In the 187 students with both urine and serum antibody tests, the concordance rate was 97.9% and kappa coefficient was 0.789 (Table 1). Four students showed discrepant results between urine and serum antibody tests (Table 1 and Table 2). Three students had negative urine antibody tests but positive serum antibody tests, and the other student was urine positive but serum negative. Of the 175 subjects with negative results for both tests, four showed serum antibody titers of ≥3.0 U/mL (negative with relatively high titers). These four subjects and the four subjects who gave discrepant results of urine and serum antibody tests were excluded from further analyses. The eight subjects with positive results for both tests and the 171 subjects with negative results for both tests (Figure 1) were considered *H. pylori* infected and non-infected, respectively.

**Table 1.** Association of urine and serum antibody tests

		Serum antibody test			Total

		Positive	Negative high titer	Negative low titer	

		(≥10.0 U/mL)	(<10.0 U/mL and ≥3.0 U/mL)	(<3.0 U/mL)	
Urine antibody test	Positive	8^a^	0	1^b^	9
	Negative	3^b^	4^b^	171^c^	178

Total		11	4	172	187

^a^Considered positive for *H. pylori* infection.

^b^These subjects were excluded from further analyses.

^c^Considered negative for *H. pylori* infection.

**Table 2.** Lists of subjects with serum antibody titers between 3.0 and 10.0 U/mL, with discrepant results of urine and serum antibody tests, and with positive results for both tests

Subjects	Gender	Urine Ab CI value	Urine Ab test	Serum Ab EV value	Serum Ab test	PG I (ng/mL)	PG II (ng/mL)	PG I to PG II ratio
Subjects with serum antibody titers <10.0 U/mL and ≥3.0 U/mL	F	0.06	−	3	−	60.2	10.4	5.8
	M	0.10	−	3	−	42.7	9.9	4.3
	M	0.05	−	3	−	48.5	9.9	4.9
	M	0.18	−	4	−	45.6	8.4	5.4

Subjects with discrepant results of urine and serum antibody tests	F	0.07	−	16	+	26	5.2	5.0
	F	0.05	−	11	+	160	71.9	2.2
	F	0.04	−	13	+	18.8	5.3	3.5
	M	1.36	+	<3	−	38.2	12.7	3.0

Subjects with positive results for both urine and serum antibody tests (Assumed as *H. pylori* infected)	M	10.08	+	67	+	38.1	11.4	3.3
	F	9.78	+	63	+	46.3	13.1	3.5
	F	1.20	+	24	+	99.9	46.0	2.2
	M	2.82	+	19	+	49.3	14.8	3.3
	M	2.51	+	36	+	62.2	19.2	3.2
	F	10.59	+	78	+	63.6	33.9	1.9
	M	9.73	+	≥100	+	61.5	30.2	2.0
	F	8.14	+	57	+	44.9	13.5	3.3

**Figure 2.**
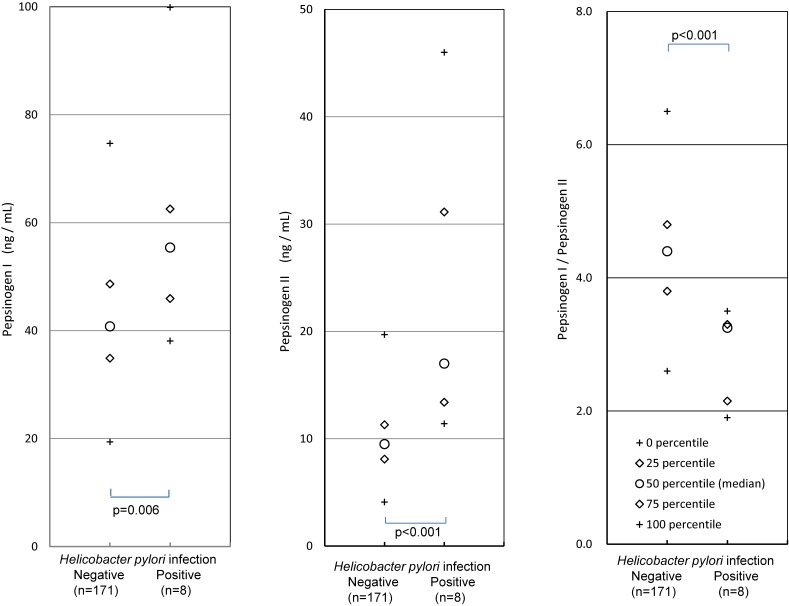
Comparison of PG values between *H. pylori* infected and non-infected subjects

In the *H. pylori* non-infected subjects, the medians of PG I, PG II, and the PG I/II ratio were 40.8 ng/mL, 9.5 ng/mL, and 4.4, respectively, whereas in the infected subjects, these values were 55.4 ng/mL, 17.0 ng/mL, and 3.3, respectively (*P*-values all <0.01; Figure 2). Percentiles of PG values in *H. pylori* non-infected subjects are shown in Table 3.

**Table 3.** Pepsinogen values in *H. pylori* non-infected 171 junior high school students

	Percentile				

	0%	25%	Median	75%	100%
PG I (ng/mL)	19.4	34.9	40.8	48.7	74.7
PG II (ng/mL)	4.1	8.1	9.5	11.3	19.7
PG I to II ratio	2.6	3.8	4.4	4.8	6.5

**Figure 3.**
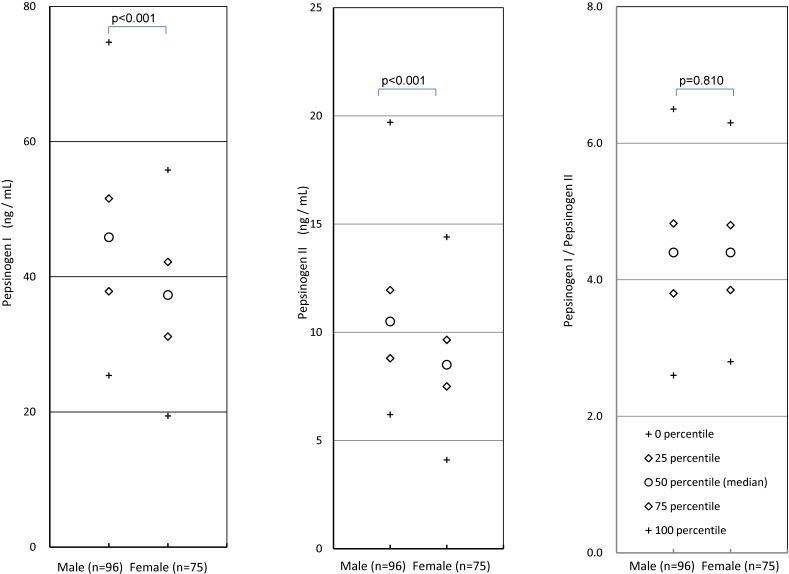
Comparison of PG values between males and females in *H. pylori* non-infected subjects

In the *H. pylori* non-infected subjects, school year (age) did not affect PG I, PG II, or the PG I/II ratio (data not shown), with *P*-values of 0.57, 0.08, and 0.11, respectively. The males showed higher PG I and PG II values than the females, whereas no gender difference was observed regarding the PG I/II ratio (Figure 3).

To evaluate the effect of excluding the eight subjects, sensitivity analyses were performed, where the four subjects with discrepant results between urine and serum tests were considered infection positive and the four subjects with serum antibody titers 3 or 4 U/mL (negative with relatively high titers) were considered infection negative. When these eight subjects were added, the medians of PG I, PG II, and the PG I/II ratio were 41.0 ng/mL, 9.5 ng/mL, and 4.4, respectively, in the *H. pylori* infected negative subjects, whereas in the infected subjects, these values were 47.8 ng/mL, 14.2 g/mL, and 3.4, respectively. Inclusion of the eight subjects slightly affected PG I and PG II in the *H. pylori* infected subjects.

## DISCUSSION

Eight junior high school students with positive results for both the urine and serum antibody tests were considered *H. pylori* infected, and 171 students with negative urine antibody results and serum antibody titers of <3.0 U/mL were considered non-infected. In the *H. pylori* non-infected subjects, the medians of PG I, PG II, and the PG I/II ratio were 40.8 ng/mL, 9.5 ng/mL, and 4.4, respectively, whereas in the infected subjects, these values were 55.4 ng/mL, 17.0 ng/mL, and 3.3, respectively (each *P* < 0.01; Figure 2). In the previous studies with asymptomatic Japanese children,^15^^,^^16^ the elevated PG I and PG II levels and the decreased PG I/II ratio in *H. pylori* infected subjects were consistent with the present study. In asymptomatic children aged 0–4 years in Chile, similar results were obtained, while the PG I/II ratio was elevated in the *H. pylori* seropositive children aged 5–9 years.^21^ In symptomatic children abroad, PG I and II were elevated and the PG I/II ratio was decreased in *H. pylori* seropositive children,^22^^,^^23^ although a few exceptional results were reported, where PG I was not affected^24^^,^^25^ or the PG I/II ratio was not affected.^26^ The elevated PG I and PG II and decreased PG I/II ratio in *H. pylori* infected children seem to be consistent, although several exceptions exist. The change in PG values in the infected children were similar to those of adult subjects without severe mucosal atrophy.^4^^,^^5^

**Figure 4.**
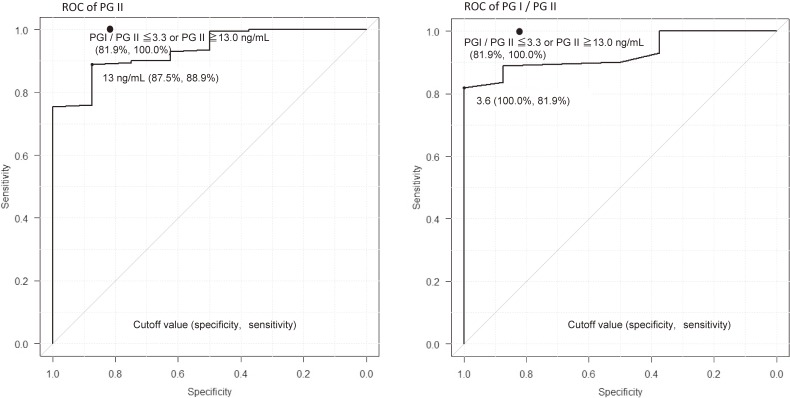
ROC (receiver operating characteristics) curves of PG II and PG I/PG II for *H. pylori* infection. The optimal criterion using both PG II and PG I/PG II is shown as closed circle (for more details see text).

PG I and PG II values can be affected by the kits that are used and the different criteria are proposed.^10^^,^^27^ One study that used the same kit as the present study reported a 96.3% sensitivity and 82.8% specificity in adult subjects when a PG II value of ≥10 ng/mL or a PG I/II ratio of ≤5.0 were considered positive for the diagnosis of *H. pylori* infection.^10^ Under that criterion, the eight *H. pylori* infected children who were diagnosed as positive in our study were considered positive, indicating 100% sensitivity, whereas 146 of the 171 non-infected children were diagnosed as positive, indicating 14.6% specificity (data not shown). These criteria for adult subjects are not appropriate for young subjects. Nonetheless, 100% sensitivity and 81.9% specificity are obtained when PG II values of ≥13 ng/mL or PG I/II ratios of ≤3.3 are used to determine *H. pylori* infection status (Figure 4), which was obtained as below. ROC of PG II indicates 13 ng/mL was the optimal cutoff value, which an infected subject did not satisfy. If the condition that the PG I/PG II ratio was ≤3.3 was added, 100% sensitivity was obtained whereas specificity decreases from 88% to 82%. Thus, PG values can be a good marker for *H. pylori* infection in junior high school students and can be used with a serum antibody test as another marker of the infection to improve the diagnostic accuracy. Nonetheless, the optimal criteria may differ from those for adults. Further study is necessary to determine the optimal criteria for practical use in junior high school students. There are gender differences regarding PG I and PG II, but not in the PG I/II ratio. The gender differences of the medians were 8.6 ng/mL for PG I and 2.0 ng/mL for PG II, whereas they were 5.4 and 0.2 ng/mL in adult subjects, respectively.^10^ Clear gender differences in PG I and PG II values may exist among junior high students, which should be considered when optimal criteria of PG values for *H. pylori* infection are determined.

There are several limitations in the present study. First, the sample size of *H. pylori* positive subjects was very small because of the low *H. pylori* prevalence in this age group in Japan. The small sample size might make the results unstable. The results of the present study regarding *H. pylori* positive subjects were consistent with other studies.^15^^,^^16^ The effect of *H. pylori* infection on PG values may be typical, and the small sample size may not have a large effect. Second, the participation rate was low. The collection of blood samples can cause pain, which may be a hurdle preventing the subjects from participating. Most subjects or their guardians did not know the *H. pylori* infection status or PG values of the subjects when they decided to participate. Therefore, the low participation rate may not affect the difference in distributions of PG values between *H. pylori* non-infected and infected subjects, between genders, or between students and adults observed in the present study. However, it may provoke a little bias on *H. pylori* prevalence because family history of *H. pylori* infection and the related disease may be a motive for the participation and intra-familial infection is thought to be the main infection route in Japan.^28^ The local government performed a *H. pylori* urine antibody screening program in 2014 for junior high school students aged 12–13 years in the area, which 355 (97%) of 366 invited students attended. The urine antibody prevalence was 5.4% and prevalence considering the results of urea breath test was 2.8–4.2% (unpublished data), which are not so different from the observed prevalence 4.5%, suggesting that selection bias may be small. Third, the students were evaluated using only urine and serum tests to identify whether a subject is *H. pylori* infected. Some reports have suggested that the antibody test is inaccurate for the diagnosis of *H. pylori* infection in children,^29^^,^^30^ but the both serum and urine antibody kits showed a good diagnostic accuracy.^17^^–^^19^ On the other hand, a strength of the present study is that both urine and serum antibody tests were performed for *H. pylori* infection diagnosis. We excluded four subjects with discrepant results and four subjects with negative high titers of serum antibody (Table 1). The exclusion decreased subjects with possible misdiagnoses and consequently may have elevated the accuracy of diagnosis.

In conclusion, PG I and PG II values were higher and the PG I/II ratio was lower in *H. pylori* infected junior high school students compared to non-infected students. In *H. pylori* non-infected students, males showed higher PG I and PG II values than females. The distributions of PG values differed from those in adults, and separate criteria must be determined for practical use in junior high school students.
